# Rapid Structure Determination of Bioactive 4″-Tetrahydrofurfuryl Macrozone Reaction Mixture Components by LC-SPE/Cryo NMR and MS

**DOI:** 10.3390/molecules26206316

**Published:** 2021-10-19

**Authors:** Iva Habinovec, Ivana Mikulandra, Lucia Ema Sekula, Jana Gašperov, Saša Kazazić, Predrag Novak

**Affiliations:** 1 Department of Chemistry, Faculty of Natural Sciences, University of Zagreb, Horvatovac 102a, HR-10000 Zagreb, Croatia; ihabinovec@chem.pmf.hr (I.H.); imikulandra@chem.pmf.hr (I.M.); lsekula@chem.pmf.hr (L.E.S.); gasperov.jana@gmail.com (J.G.); 2 Ruđer Bošković Institute, Bijenička cesta 54, HR-10000 Zagreb, Croatia; Sasa.Kazazic@irb.hr

**Keywords:** azithromycin conjugates, macrozones, HPLC-SPE/cryo NMR, MS, structure determination

## Abstract

LC-SPE/cryo NMR and MS methodologies have been developed and employed for a rapid structure determination of 4″-tetrahydrofurfuryl macrozone reaction mixture components. Macrozones, novel conjugates of azithromycin, and thiosemicarbazones have shown very good in vitro antibacterial activities against susceptible and some resistant bacterial strains and are promising agents for further development. The post-column multiple trapping of the chromatographically separated reaction mixture components on the SPE cartridges increased the sensitivity and together with cryogenically cooled NMR probe made it possible to identify and structurally characterize main 4″-tetrahydrofurfuryl macrozone reaction mixture compounds including those present at very low concentration level. This approach has several advantages over a classical off-line procedure, efficiency and low solvent consumption being the two most important ones. All identified components were process-related. It has been demonstrated that two different kinds of compounds with respect to structure were identified, i.e., macrolide-related and thiosemicarbazone-related ones. This methodology can serve as a platform for reliable and effective macrolides reaction components structure profiling, serving as both isolation and identification tools.

## 1. Introduction

**Scheme 1:**
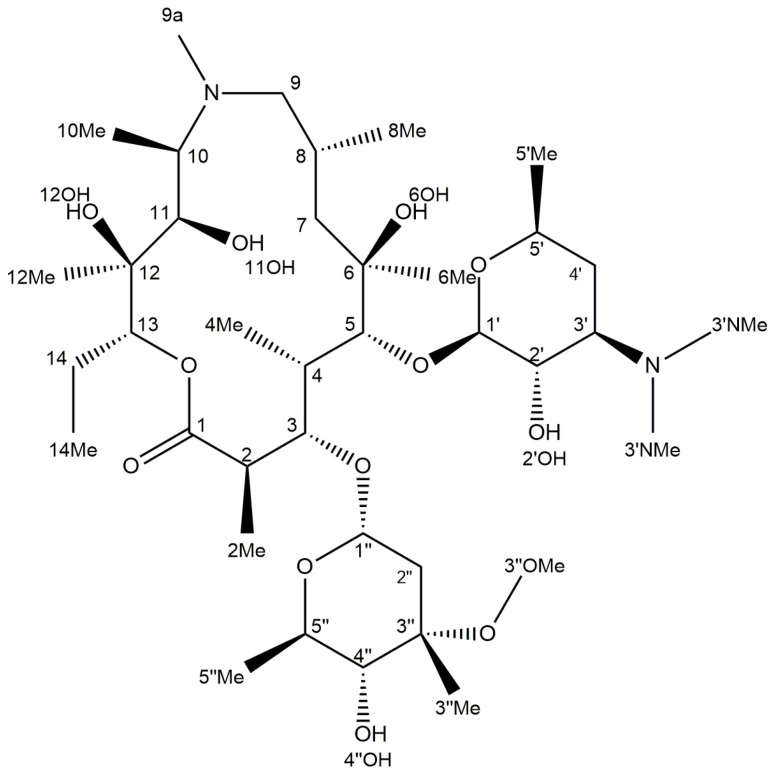
The structure of azithromycin and the atom numbering.

Azithromycin belongs to second generation macrolide antibiotics with a broad spectrum of antibacterial activity [1,2]. It is very efficient and well-tolerated anti-infective agent widely prescribed for treating upper and lower respiratory tract infections. Azithromycin is a semi-synthetic derivative of 14-membered macrolide erythromycin, but compared to erythromycin, azithromycin possesses improved pharmacokinetics, with high cell accumulation, metabolic stability, and tolerability. It shows beneficial effects in chronic inflammatory diseases and malaria as well. As an antibiotic it shares the same mode of action as other 14- and 16-membered macrolides [3] by binding to the bacterial ribosomal 23S rRNA in domain V at the peptidyl transferase region and sterically blocking the nascent peptide exit tunnel thus inhibiting the bacterial protein synthesis. Structurally, azithromycin consists of 15-membered macrocyclic lactone ring to which desosamine and cladinose sugar units are attached at positions 3 and 5, respectively (Scheme 1). Crystallography [4,5,6] and NMR spectroscopy [7,8,9,10] have revealed key structural features of macrolide antibiotic interactions with ribosome and other macromolecular receptors that serve as platform for design of novel bioactive macrolide compounds.

However, rapidly emerging bacterial resistance to existing antibiotics including macrolides in the last couple of decades presents a serious threat to human health and novel bioactive compounds are urgently needed. There are two main mechanisms by which bacteria resist macrolides a) by modifications of binding sites and b) by efflux pump activity [2,11].

We have recently designed and prepared novel conjugates of azithromycin, the macrozones, in order to overcome the bacterial resistance mechanisms [12]. Some azithromycin conjugates have already proven useful to treat pathogenic bacteria [13,14,15,16,17,18,19,20,21,22,23,24]. The macrozones, hybrid compounds of azithromycin and thiosemicarbazones, have shown excellent antibacterial activities against susceptible and resistant strains [12].

Here we present the utility of using LC-SPE/cryo-NMR and MS approach for fast and efficient characterization of 4″-tetrahydrofurfuryl macrozone reaction mixture to get insights into reaction components identity and structure. Modern and sophisticated hyphenated techniques such as LC-NMR, LC-MS and others have frequently been used to analyze complex mixtures without initial separation of the constituents that have enabled rapid screening of pharmaceutical and biological samples [25,26,27,28,29,30,31,32,33]. Initial sensitivity limitations of LC-NMR have in the past two decades been largely alleviated by technological developments that include cryo-platforms, capillary NMR with micro-coil probes and coupling of solid phase extraction system (SPE) into an LC-SPE-NMR set up [30,34,35,36,37,38]. The latter methodology has been widely used for analyzing natural products, metabolites and drug impurities but has less frequently been applied for monitoring chemical reactions and structural identification of the reaction products [39,40,41,42,43]. In the present work, we applied LC-SPE/cryo-NMR in combination with MS for rapid identification and structural characterization of bioactive macrozone reaction components.

## 2. Results and Discussion

**Figure 1:**
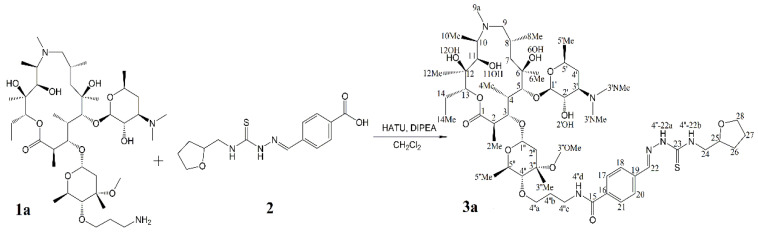
The final reaction step for preparing 4″-tetrahydrofurfuryl macrozone **3a** and the atom numbering.

The preparation of amino propyl derivative of azithromycin 1a and tetrahydrofurfuryl thiosemicarbazone acid 2 were reported in the previous paper [12]. The macrozone 3a (Figure 1) showed very good in vitro antibacterial activities against susceptible bacterial strains such as *S. pneumoniae* and *S. pyogenes* strains comparable to that of azithromycin. It exhibited four times better activity than azithromycin against efflux resistant *S. pneumoniae* and *E. faecalis* strains and also against efflux resistant *S. aureus* to which azithromycin is inactive. This compound shows potential for derivatizations to further improve activity against resistant bacteria.

### 2.1. Optimization of Chromatographic Separation

**Figure 2:**
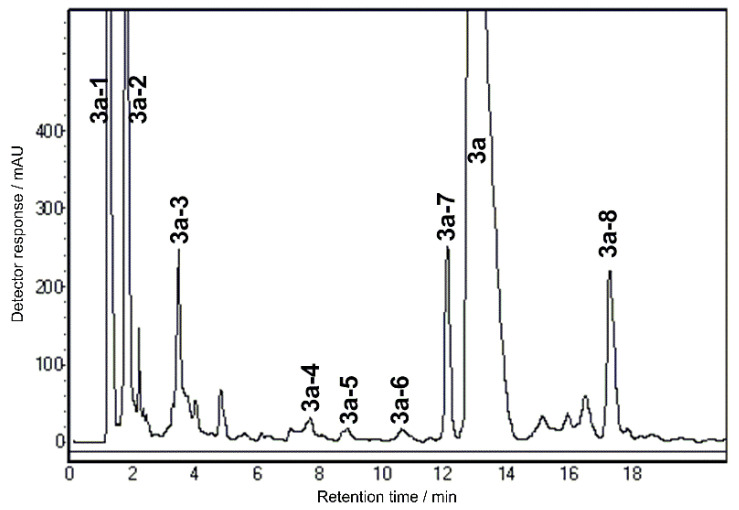
LC-DAD chromatogram of the macrozone **3a** reaction mixture with assigned main components.

The first step in the analysis of the components present in the reaction mixture was to obtain chromatographic separation conditions that provide high quality and well-resolved chromatographic peaks. Optimised chromatographic separation of the individual reaction mixture components was achieved on Waters XBridge Phenyl column and Poroshell 120 EC-C18 column, respectively. Since retention times of the chromatographic peaks were quite shorter on a phenyl column and peak resolution was satisfying for LC-SPE trapping, Waters XBridge Phenyl column was used for further analysis. The chromatogram is displayed in Figure 2.

### 2.2. SPE Preconcentration

In order to obtain a sufficient amount of a compound for 2D NMR experiments, it is necessary to obtain high extraction efficiency of the individual peaks on SPE-cartridges. Therefore, extraction efficiency of macrozone reaction mixture components on different SPE-stationary phases was investigated. For that purpose, HySphere method development cartridge tray, consisted of C2, C8, CN, Resin SH, Resin GP, MM cation and MM anion SPE-stationary phases, was used. Compounds were trapped several times on each SPE-cartridge at the make-up flow rate of 3 mL min^−1^. After the elution of samples into 3 mm NMR tubes, ^1^H NMR spectra were recorded and signal-to-noise ratio was calculated. Results showed that most of the reaction mixture components were well retained at C18 SPE-cartridges. Furthermore, the influence of make-up flow rate on extraction efficiency was also evaluated. Make-up flow rate of 1.5 mL min^−1^ was optimal for the most cases, while more polar compounds were better retained on SPE-cartridges using the higher make-up flow rates (up to 3.0 mL min^−1^).

### 2.3. Identification of Components by Cryo NMR and MS

**Figure 3:**
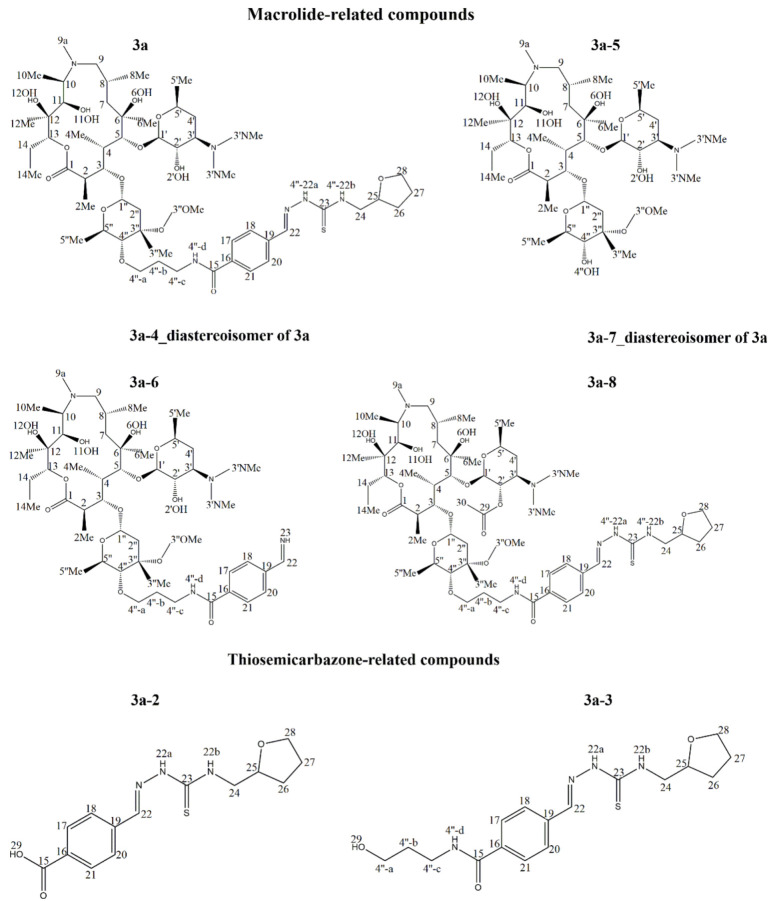
The stuctures of **3a** and its reaction mixture components. The compounds **3a-4** and **3a-7** are diastereoisomers of **3a**.

The main compound, the macrozone 3a, was readily identified at 13.2 min retention time with 34.6% content. Analysis of proton chemical shifts and correlation peaks in COSY, HSQC and HMBC spectra (Supplementary Material Figure S1a–d) confirmed the structure of 3a as shown in Figure 3. The assignments of proton and carbon chemical shifts are given in Table 1.

**Figure 4:**
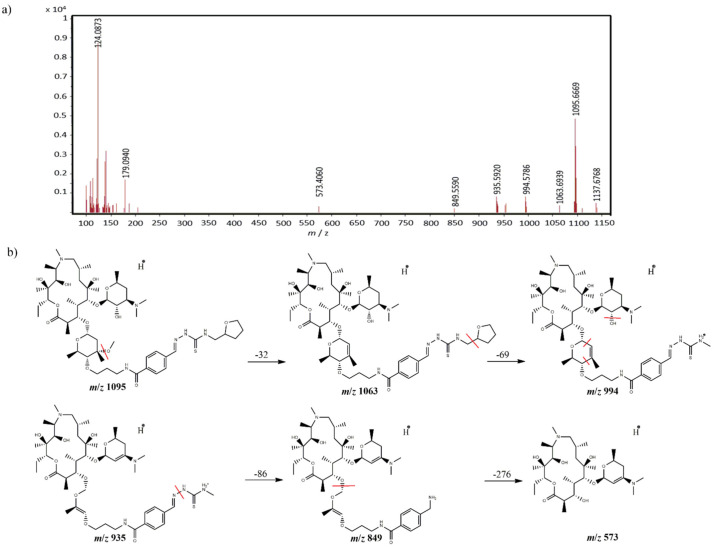
(**a**) MS/MS spectrum and (**b**) fragmentation pattern of 4″-tetrahydrofurfuryl macrozone **3a**.

The molecular ion peak (*m*/*z* 1095.6) and the fragmentation pattern depicted in Figure 4 are in accordance with the proposed structure.

As can be seen in Figure 2 the reactant 2 and the carboxylic acid activation reagent HATU (hexafluorophosphate azabenzotriazole tetramethyl uronium) used in the reaction elute first (3a-1 and 3a-2 in the chromatogram, respectively), which is expected since those are the most polar components. NMR spectra of the compounds 3a-1 and 3a-2 are given in Supplementary (Figures S2 and S3). The proton and MS spectra of the compound 3a-3 with the peak area of 1.4% clearly shows that macrolactone ring and sugar units are missing and that this compound is related to thiosemicarbazone constituent. The molecular ion was identified at *m*/*z* 364.3 (Figure S4). Based on this data, we propose the structure as depicted in Figure 3. The reaction component 3a-4 with the content of 0.12% displayed the same molecular ion peak at *m*/*z* 1095.5 as the main compound reflecting the presence of a 3a diastereoisomer. This compound exhibits similar ^1^H (Figure S5) and MS patterns as the main compound confirming a diastereoisomeric structure. Even with using higher make-up flow rates, the final concentration was not sufficient for recording a reliable NOESY spectrum to possibly identify a stereoisomeric center.

**Figure 5:**
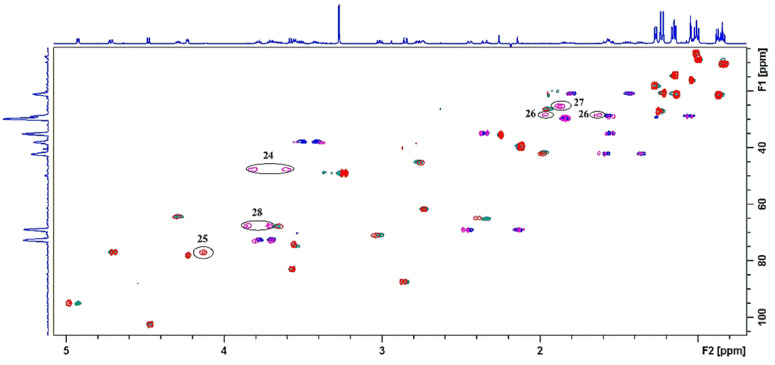
Superimposed HSQC spectra of **3a** (red and pink contours) and **3a-6** (green and blue contours) with characteristic thioureido-furfuryl cross peaks highlighted.

A low-level component **3a-5**, present with the amount of only 0.3% at the retention time of 9.0 minutes was unambiguously identified as azithromycin, by its ^1^H, COSY and MS spectra (Figure S6). A close inspection of ^1^H spectrum and connectivities in COSY, HSQC and HMBC spectra of **3a-6** (0.59%, 10.6 min) revealed that thioureido-furfuryl moiety was not present in the molecule. In Figure 5, it is clearly seen from the overlapped HSQC spectra that cross peaks (circled in black) indicative of thioureido-furfuryl side chain present in the spectrum of **3a** are missing in the spectrum of **3a-6**. The molecular ion peak observed at *m*/*z* 938.7 (Figure S7d) corroborates this observation. All other cross peaks characteristic of the main compound were present in the spectra. These findings pointed toward the structure depicted in Figure 3.

The component **3a-7** present at 2.5% level at the retention of 12.1 min displayed the same molecular ion *m*/*z* as the main compound **3a** (*m*/*z* 1095.6) implying the presence of an additional diastereoisomer. Analysis of NMR spectra has led to straightforward assignments of proton and carbon chemical shifts similar to **3a**. (Table S1, Figure S8a–e). However, the trapping efficiency was not good enough, and the final concentration was again too low for unambiguous NOESY analysis.

The analysis of ^1^H chemical shifts and COSY, HSQC, and HMBC cross peaks of **3a-8** (4.4%, 17.3 min) revealed the same patterns found for **3a** except for an additional methyl, H30, and carbonyl group C29 (Figure 3) observed at 39.5 and 175.1 ppm, respectively, and displaying a common HMBC correlation peak. Other HMBC correlations unambigously pointed toward the 2′-methyl ester of **3a** whose structure is displayed in Figure 3. This compound is an intermediate in the synthesis of amino propyl derivative 1a [12]. The spectra are given in Supplementary Materials (Figure S9a–d).

In conclusion, we have shown here that LC-SPE/cryo NMR methodology in combination with MS can successfully be applied for rapid screening of bioactive macrozone reaction mixture and straightforward identification and structure characterization of the main components.

## 3. Materials and Methods

### 3.1. Reagents

4″-tetrahydrofurfuryl macrozone was synthesized in our laboratory according to the procedure reported in a previously published article [12]. HPLC-grade acetonitrile was purchased from Fisher Scientific (Loughborough, UK). HPLC-grade ammonium bicarbonate was obtained from Sigma-Aldrich (St. Louis, MO, USA). Deionized water was produced by Millipore Milli-Q Advantage A10 purification system (Molsheim, France). MS-grade acetonitrile was purchased from Carlo Erba (Val de Reuil, France). Deuterated acetonitrile (99.8%-D) was procured from Euriso-Top SAS (Saint-Aubin Cedex, France).

### 3.2. Sample Preparation

The reaction mixture of 4″-tetrahydrofurfuryl-macrozone (**3a**) was evaporated to dryness and stored at 4 °C. Prior to LC-SPE analysis samples were dissolved in acetonitrile at concentration of 14.3 mg mL^−1^. For the MS analysis dry extracts of isolated compounds were prepared at concentration ranging from 0.11 µg mL^−1^ to 0.52 µg mL^−1^ by dissolving in MS-grade acetonitrile.

### 3.3. Liquid Chromatography

Chromatographic analysis was performed on Agilent 1260 Infinity HPLC system consisting of quaternary pump G1311B, an autosampler G1329B, thermostated column compartment G1316A and a photo diode array detector G1315D. The chromatographic data were collected by OpenLab CDS Chemstation A.01.08.108. To optimize chromatographic separation of macrozone reaction mixture components several C18 chromatographic columns and phenyl column were used, including the Ascentis Express C18 (100 × 4.6 mm; 2.7 µm, Sigma-Aldrich, St. Louis, MO, USA), Kinetex C18 (150 × 4.6 mm; 5 µm, Phenomenex, Torrance, CA, USA), Zorbax SB-C18 (150 × 4.6 mm; 3.5 µm, Agilent, Santa Clara, CA, USA), Poroshell 120 EC-C18 (250 × 4.6 mm; 4 µm, Agilent, USA), Hypersil ODS (150 × 4.6 mm; 5 µm, Agilent, Santa Clara, CA, USA), XBridge C18 (150 × 4.6 mm; 3.5 µm, Waters Corporation, Milford, MA, USA) and XBridge Phenyl (150 × 4.6 mm; 3.5 µm, Waters Corporation, Milford, MA, USA). The best chromatographic separation was achieved on Waters XBridge Phenyl chromatographic column at 25 °C using a gradient elution with mobile phase consisting of (A) acetonitrile and (B) ammonium bicarbonate solution (10 mM) corrected to pH 10 with ammonia solution. Gradient elution program with a flow rate of 1.000 ml min^−1^ was as follows: 0–20 min (50% A–68% A), 20‒21 min (68% A–100% A), 21–30 min (100% A), 30‒30.1 min (100% A–50% A), 30.1–35 min (50% A). Injection volume was 15 µL and detection wavelength was 210 nm.

### 3.4. On-line HPLC-SPE

Post-column solid-phase extraction of the macrozone reaction mixture components was performed on Prospekt 2 SPE unit (Spark Holland, Netherland). Analytes were trapped on HySphere C18 HD SPE-cartridges (2 mm × 10 mm) using the multi-trapping mode. Threshold absorbance levels for analyte trapping were set at 210 nm. Sample injection volume was 15 µL. The post-column diluent (make-up solvent) was solution B with a flow rate of 1.5 ml min^−1^. After the peak trapping, SPE-cartridges were dried in nitrogen stream for 59 min. Components were eluted from SPE-cartridges to 3 mm NMR tubes with 150 µL of acetonitrile-d3 and NMR spectra were recorded. LC-SPE data were collected and analyzed with HyStar 3.2 software.

### 3.5. NMR Spectroscopy

NMR spectra with NOESY-type solvent suppression module were recorded on Bruker Avance III HD 400 MHz and Bruker Avance NEO 600 MHz spectrometers equipped with broadband observed (BBO) Prodigy and inverse TCI Prodigy cryoprobes, respectively, and z-gradient accessories. After LC separation and peak trapping on the SPE unit, the separated components were dissolved in acetonitrile-d3 and measured in 3 mm tubes with MATCH inserts for adapting the tubes to 5 mm Bruker spinners. All experiments were carried out at 298 K using TMS as an internal standard.

Proton spectra were measured with 128–512 scans, depending on the concentration of extracted components. Spectral width was 11,904 Hz and a digital resolution was 1.45 Hz per point. In the gCOSY experiment, 2048 points in the f2 dimension and 256 increments in the f1 dimension were used. For each increment, 6–32 scans and the spectral width of 9615 Hz were applied. Digital resolution was 9.39 Hz and 75.12 Hz per point in f2 and f1 dimensions, respectively.

The gHSQC spectra were acquired with 20–150 scans. In gHSQC, experiment spectral width was 9615 Hz in f2 and 27,166 Hz in f1 dimension. 4K data points were applied in the time domain and for each data set 256 increments were collected for gHSQC, as well as for gHMBC spectra. The gHMBC spectra were acquired with 40–160 scans using the spectral width of 9090 Hz in f2 domain and 33,204 Hz in f1 domain.

### 3.6. MS Analysis

Mass spectra were recorded on an amaZon ETD ion trap mass spectrometer (Bruker Daltonik, Bremen, Germany) equipped with the standard ESI ion source. Nitrogen was used as a drying and nebulizing gas. Nebulizer pressure was 8 psi, drying gas flow rate was 5 L min^−1^, drying gas temperature was 200 °C. For the mass spectrometry analysis dry extracts of LC isolated fractions were prepared at a concentration ranging from 0.11 mg mL^−1^ to 0.52 mg mL^−1^ by dissolving them in 1 mL of MS-grade acetonitrile. The samples were further diluted 1000 times in electrospray solution (acetonitrile/water with 0.1% formic acid, 50/50, *v:v*). Prepared samples were infused into the ESI source of the mass spectrometer by a syringe pump at a flow rate of 1 μL min^−1^. The mass spectrometer was operated in positive polarity mode, the potential on the capillary cap was 4500 V. Helium was used as the collision gas. Full scan ESI-MS spectra were obtained in a positive ion acquisition mode ranging from 100 *m*/*z* to 1500 *m*/*z*. Tandem mass spectra were recorded by performing collision-induced dissociation (CID) type of fragmentation. Parent ions are isolated in ±2 Da wide *m*/*z* window and fragmented by applying 0.5 V of collision energy.

## Figures and Tables

**Table 1:** ^1^H and ^13^C chemical shifs of 4″-tetrahydrofurfuryl macrozone **3a**.

Compound	3a				
Atom	*δ*(^1^H)/ppm	*δ*(^13^C)/ppm	Atom	*δ*(^1^H)/ppm	*δ*(^13^C)/ppm
**1**	-	178.98	**22**	7.97	141.6
**2**	2.76	45.3	**4″-22a**	-^a^	-
**2Me**	1.15	14.66	**4″-22b**	8.00	-
**3**	4.23	78.15	**23**	-	178.03
**4**	1.99	42.1	**24**	3.83; 3.60	47.8
**4Me**	1.00	8.99	**25**	4.14	77.1
**5**	3.57	83.1	**26**	1.97; 1.63	28.44
**6**	-	74.0	**27**	1.91	25.34
**6Me**	1.26	27.27	**28**	3.86; 3.72	67.6
**6OH**	-^a^	-	**1′**	4.47	102.6
**7eq; 7ax**	1.61; 1.37	42.1	**2′**	3.05	71.1
**8**	1.98	26.4	**2′OH**	-^a^	-
**8Me**	0.87	21.51	**3′**	2.40	65.14
**9eq; 9ax**	2.48; 2.14	69.12	**3′NMe_2_**	2.12	39.7
**9a-N**	2.25	35.6	**4′eq; 4′ax**	1.55; 1.07	29.13
**10**	2.76	62.1	**5′**	3.66	67.9
**10Me**	1.02	6.55	**5′Me**	1.14	21.24
**11**	3.66	67.9	**1″**	4.98	95.1
**11OH**	-^a^	-	**2″eq; 2″ax**	2.34; 1.56	34.9
**12**	-	74.4	**3″**	-	73.3
**12Me**	1.05	16.13	**3″Me**	1.21	20.92
**12OH**	-^a^	-	**3″OMe**	3.26	49.2
**13**	4.70	77.2	**4″**	2.87	87.5
**14eq; 14ax**	1.82; 1.44	21.0	**4″-a**	3.85; 3.73	73.5
**14Me**	0.84	10.65	**4″-b**	1.84	29.6
**15**	-	166.02	**4″-c**	3.55; 3.41	38.2
**16**	-	135.74	**4″-d**	7.50	-
**17; 21**	7.85	127.53	**5″**	4.30	64.4
**18; 20**	7.81	127.24	**5″Me**	1.28	18.18
**19**	-	136.75			

^-a^ not assigned due to a H/D exchange.

## Data Availability

The data presented in this study are available within the article and in Supplementary Materials.

## Supplementary Materials

The following are available online, Figure S1: (a) proton spectrum, (b) COSY spectrum, (c) HSQC spectrum and (d) HMBC spectrum of the 4″-tetrahydrofurfuryl macrozone **3a**; Figure S2: (a) proton spectrum, (b) COSY spectrum, (c) HSQC spectrum and (d) HMBC spectrum of the compound **3a-1** (HATU).; Figure S3: (a) proton spectrum, (b) COSY spectrum, (c) HSQC spectrum and (d) HMBC spectrum of the compound **3a-2** (reactant 2).; Figure S4: (a) proton spectrum of the **3a-3** (signals from solvent impurities are marked with asterisk* and signals from ethanol are marked with double asterisk**), (b) MS spectrum of the **3a-3** and (c) overlaid proton spectra of the **3a**, **3a-2** and **3a-3**; Figure S5: (a) overlaid proton spectra of the compounds **3a** and **3a-4** (signals from solvent impurities are marked with asterisk*), (b) COSY spectrum and (c) MS spectrum of the compound **3a-4**.; Figure S6. (a) proton spectrum of the compound **3a-5**, (b) COSY spectrum of the compound **3a-5** and (c) MS spectrum of the compound **3a-5**, Figure S7. (a) proton spectrum, (b) HSQC spectrum, (c) HMBC spectrum and (d) MS spectrum of the compound **3a-6**, Figure S8. (a) overlaid proton spectra of the **3a-7** and **3a**, (b) COSY spectrum, (c) overlaid HSQC spectrum of the **3a-7** and **3a** (blue and green contours belong to the **3a-7**, while red and pink contours belong to the **3a**; signals from unknown impurities are marked with asterisk*), (d) HMBC spectrum and (e) MS spectrum of the compound **3a-7**, Figure S9. (a) proton spectrum, (b) COSY spectrum, (c) HSQC spectrum, (d) HMBC spectrum of the compound **3a-8**. HMBC correlation of a methyl group protons H30 with a carbonyl group C29 is marked in black, Table S1: ^1^H and ^13^C chemical shifs of compound **3a-7**.

## Sample Availability

Samples of the compounds are available from the authors.
