# Supplementary material for: Rapid Structure Determination of Bioactive 4″-Tetrahydrofurfuryl Macrozone Reaction Mixture Components by LC-SPE/Cryo NMR and MS
Source: Molecules. 2021 Oct 19;26(20):6316. doi: 10.3390/molecules26206316 (PMC8537197; doi:10.3390/molecules26206316)
Supplement: Supplementary file 1 [file molecules-26-06316-s001.zip › molecules-1394943-supplementary.pdf]

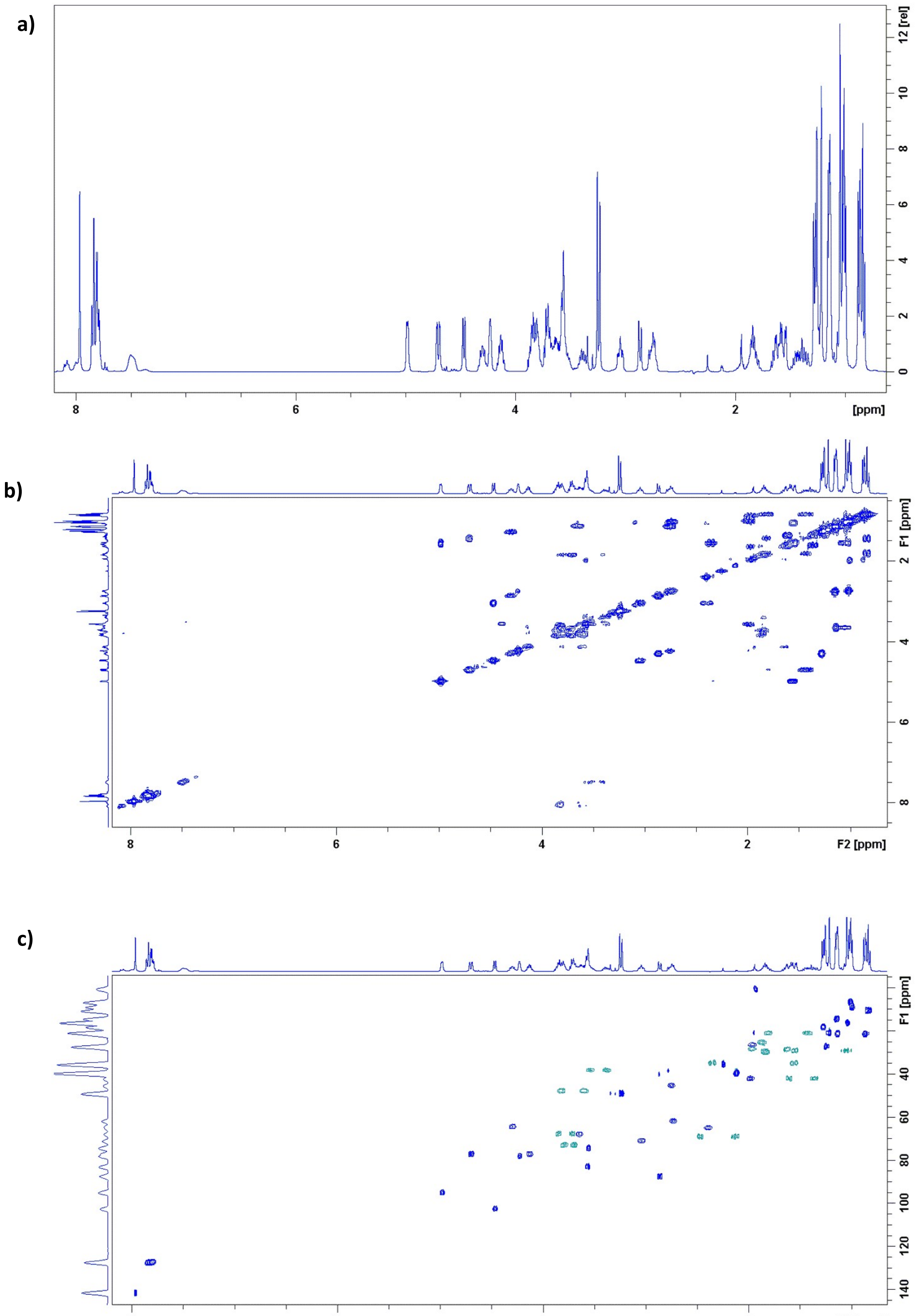
a)
12 [rel]
10
8
6
4
2
0
8
6
4
2
[ppm]
b)
F1 [ppm]
2
4
6
8
8
6
4
2
F2 [ppm]
c)
F1 [ppm]
20
40
60
80
100
120
140
8
6
4
2
F2 [ppm]

d)

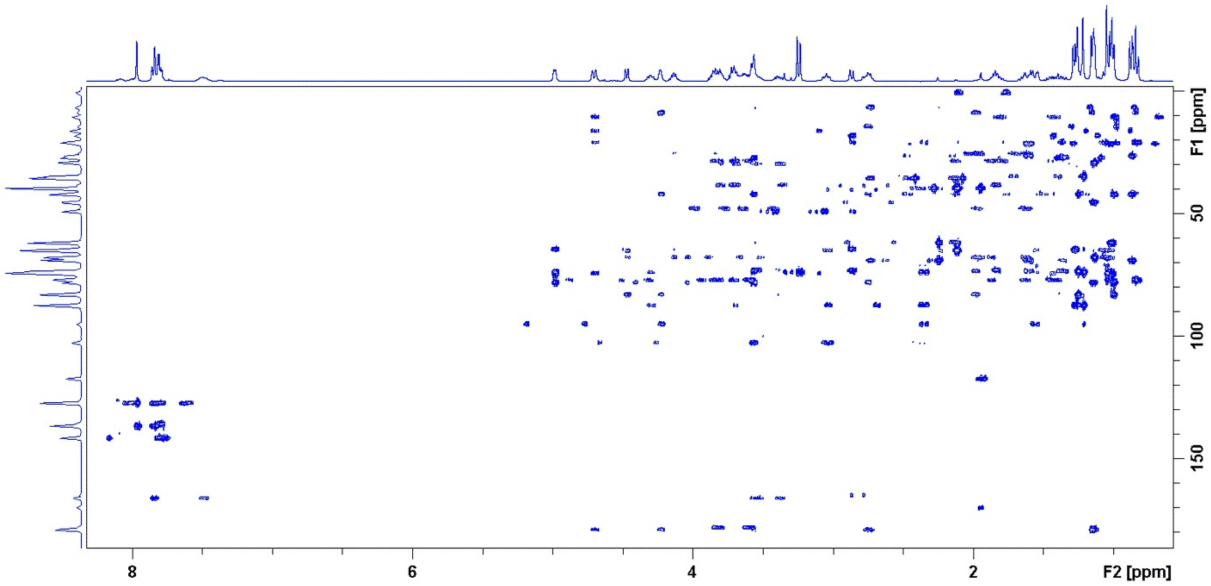

Figure S1. a) proton spectrum, **b)** COSY spectrum, **c)** HSQC spectrum and **d)** HMBC spectrum of the 4′′-tetrahydrofurfuryl macrozone **3a.**

a)

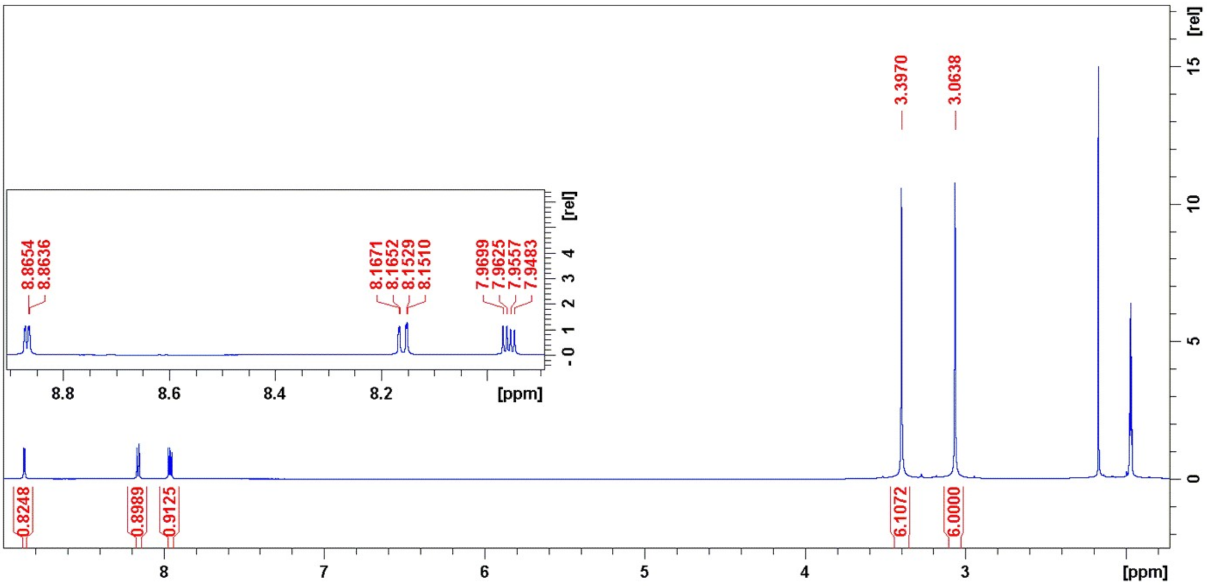
8.8654
8.8636
8.1671
8.1652
8.1529
8.1510
7.9699
7.9625
7.9557
7.9483
[rel]
[ppm]
3.3970
3.0638
0.8248
0.8989
0.9125
6.1072
6.0000
[rel]
[ppm]

b)

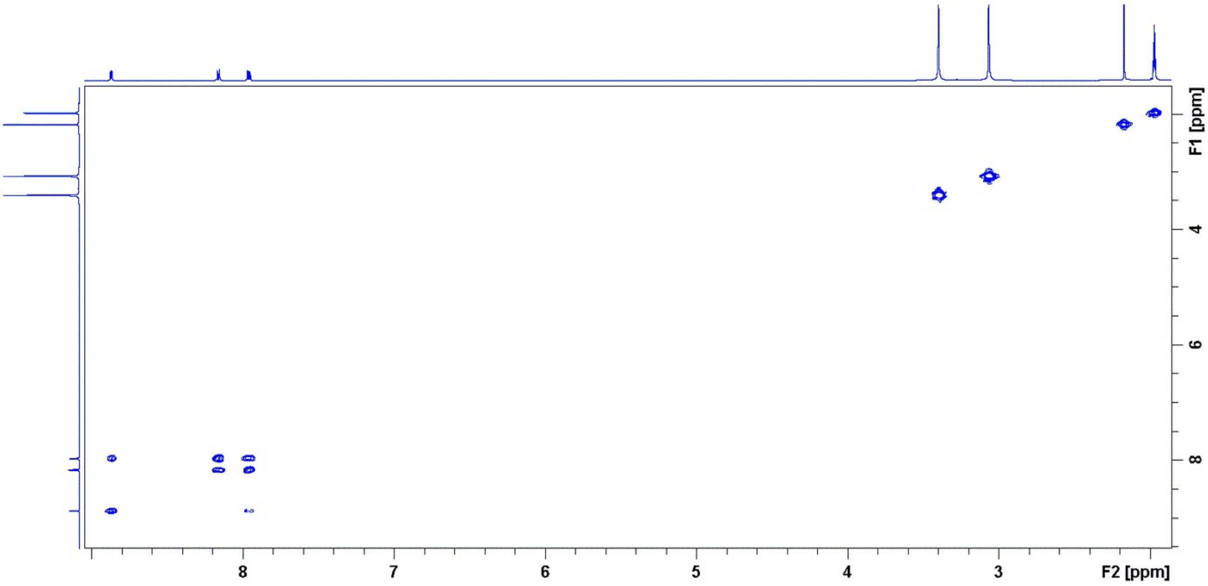
F1 [ppm]
F2 [ppm]

c)

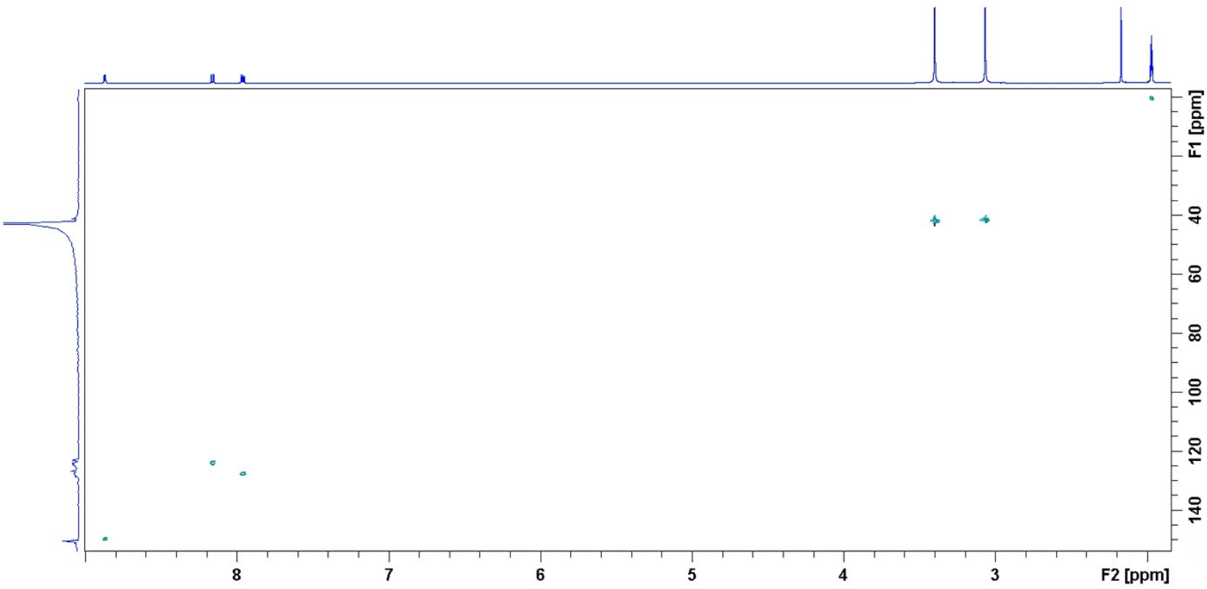
F1 [ppm]
F2 [ppm]

d)

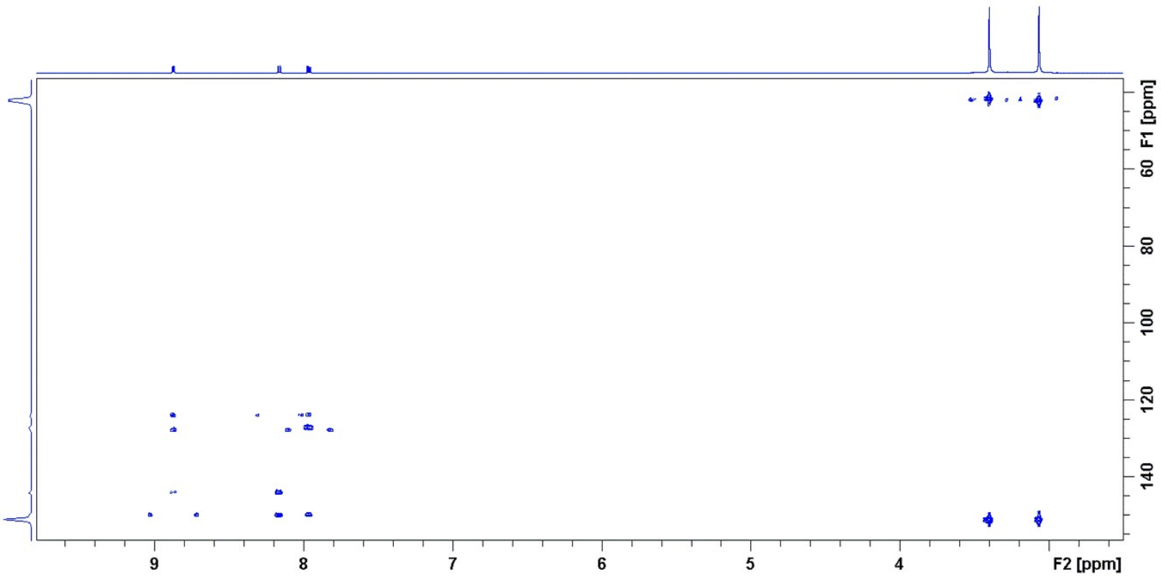

Figure S2. a) proton spectrum, b) COSY spectrum, c) HSQC spectrum and d) HMBC spectrum of the compound **3a-1 (HATU).**

a)

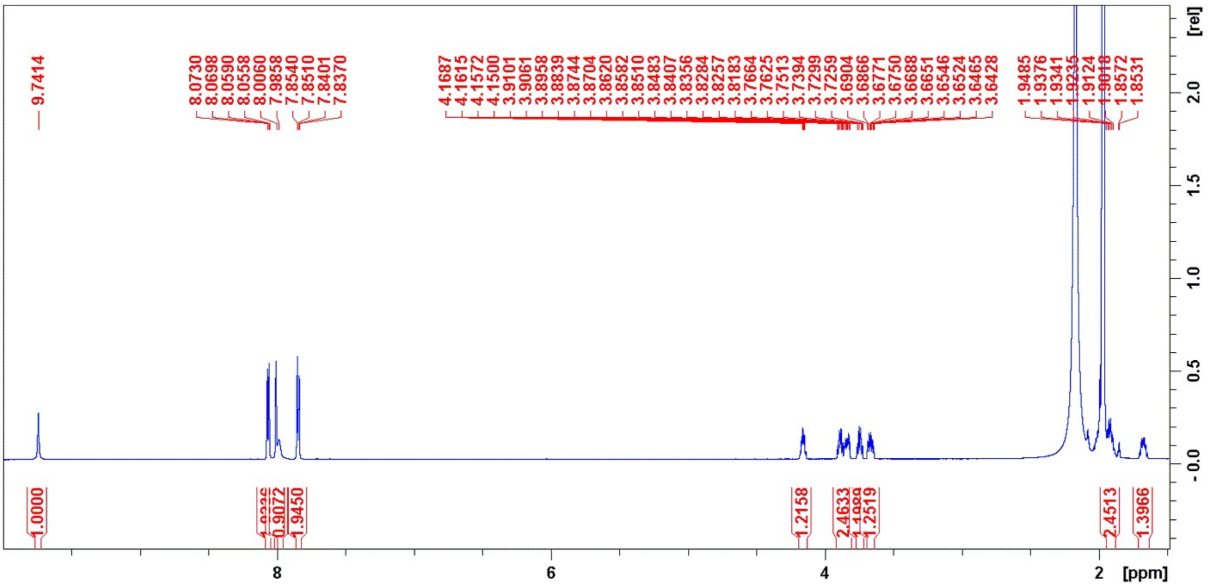

9.7414
8.0730
8.0698
8.0590
8.0558
8.0060
7.9858
7.8540
7.8510
7.8401
7.8370
4.1687
4.1615
4.1572
4.1500
3.9101
3.9061
3.8958
3.8839
3.8744
3.8704
3.8620
3.8582
3.8510
3.8483
3.8407
3.8356
3.8284
3.8257
3.8183
3.7664
3.7625
3.7513
3.7394
3.7299
3.7259
3.6904
3.6866
3.6771
3.6750
3.6688
3.6651
3.6546
3.6524
3.6465
3.6428
1.9485
1.9376
1.9341
1.9235
1.9124
1.9018
1.8572
1.8531
[rel]
2.0
1.5
1.0
0.5
-0.0
1.0000
0.9072
1.9450
1.2158
2.4633
1.2519
2.4513
1.3966
8
6
4
2
[ppm]

b)

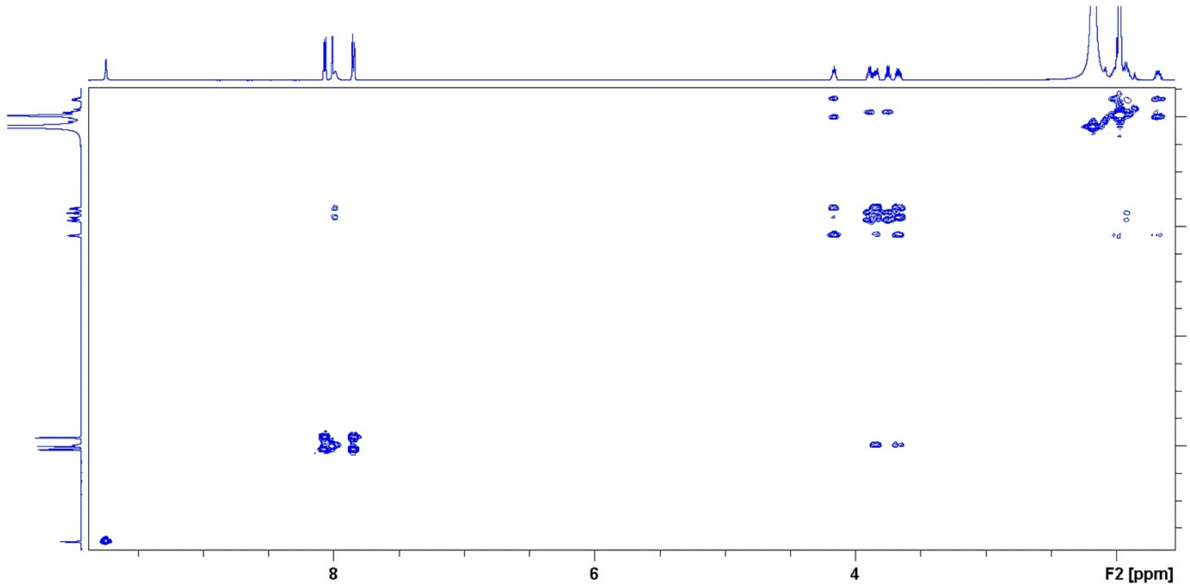

F1 [ppm]
4
6
8
8
6
4
F2 [ppm]

c)

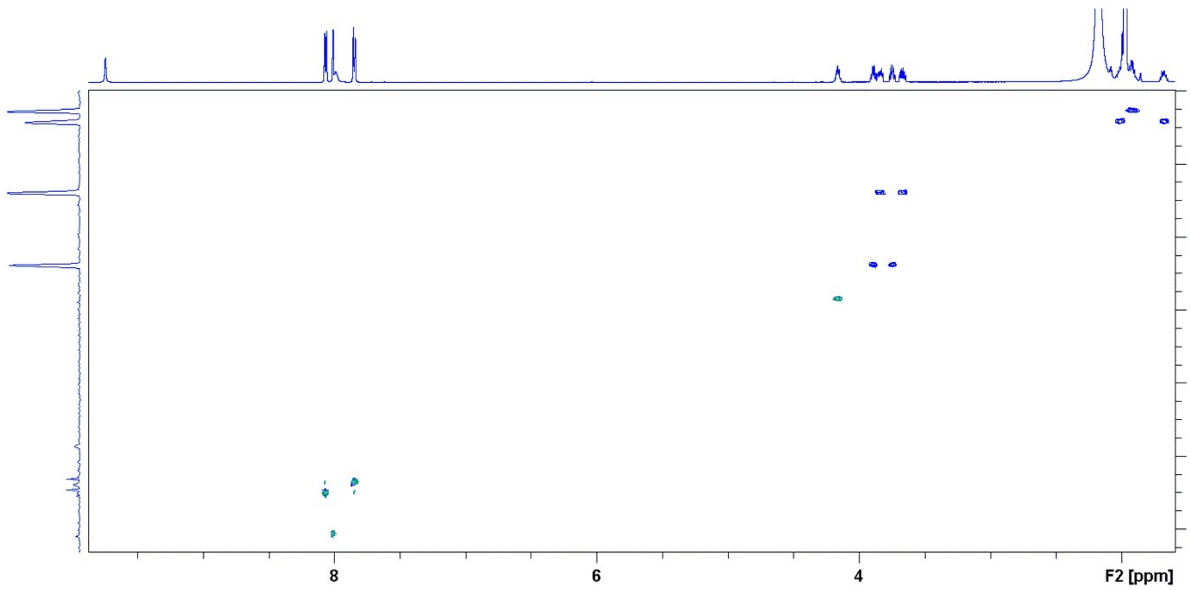

F1 [ppm]
60
80
100
120
140
8
6
4
F2 [ppm]

d)

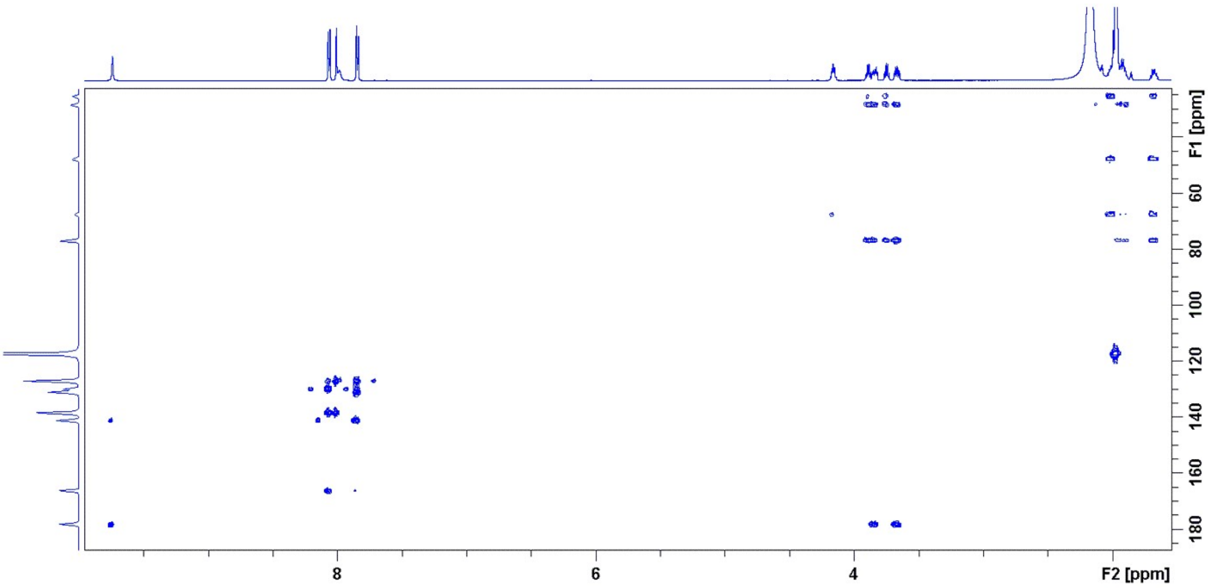

Figure S3. a) proton spectrum, b) COSY spectrum, c) HSQC spectrum and d) HMBC spectrum of the compound **3a-2 (reactant 2).**

a)

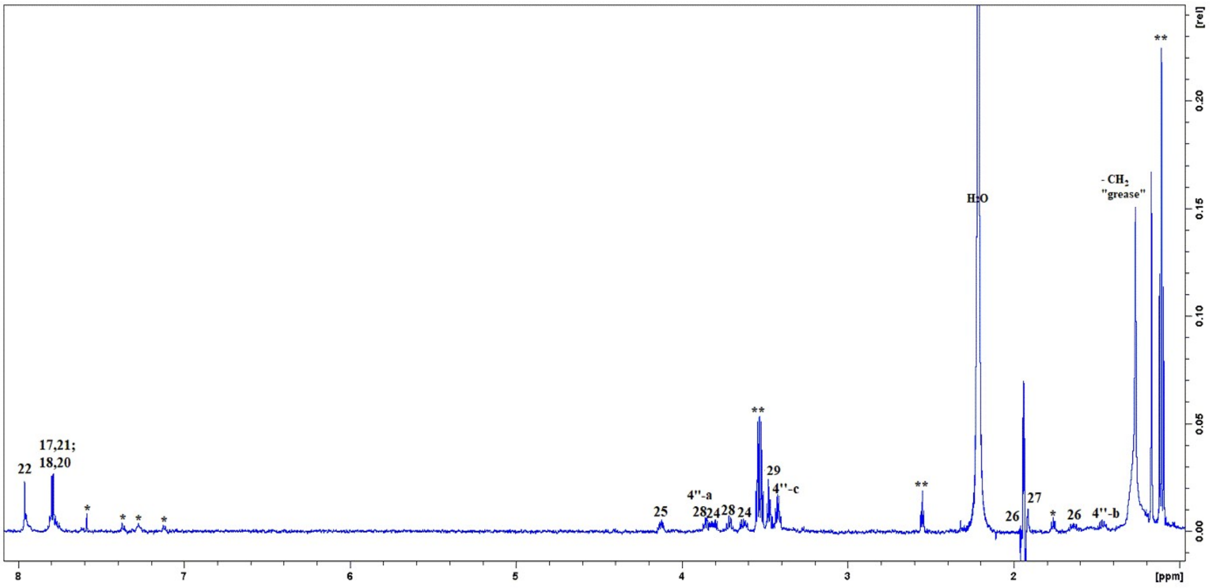

b)

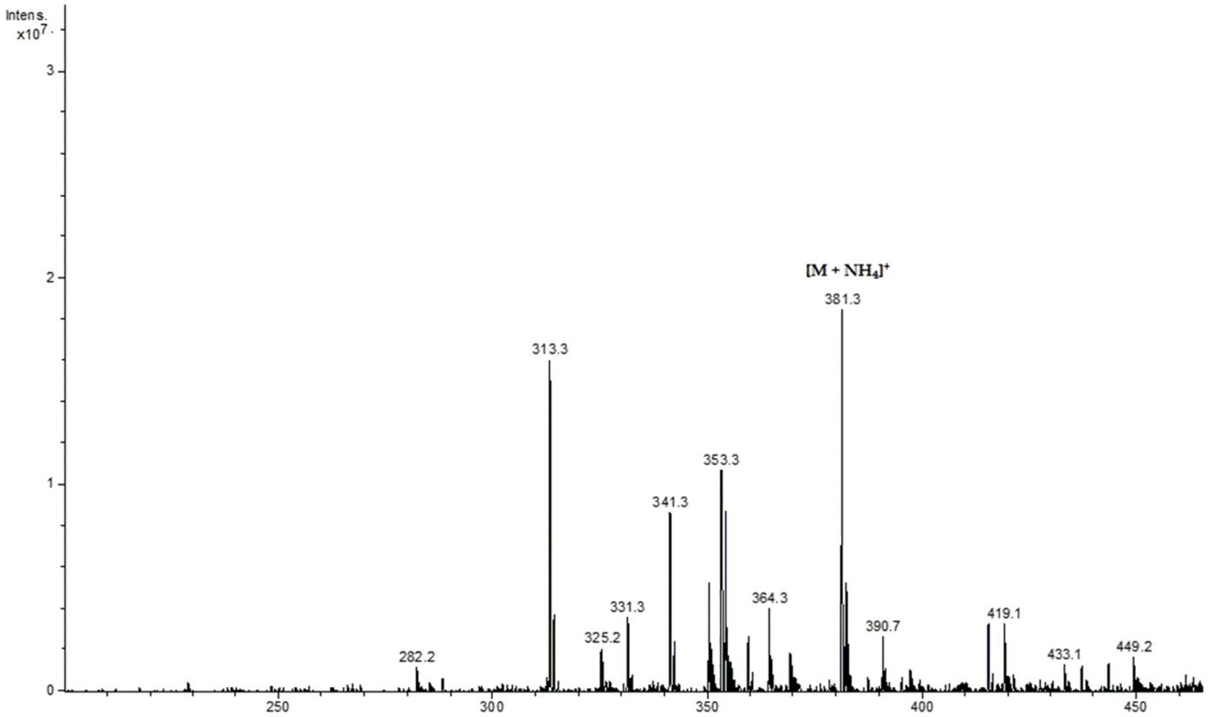

c)

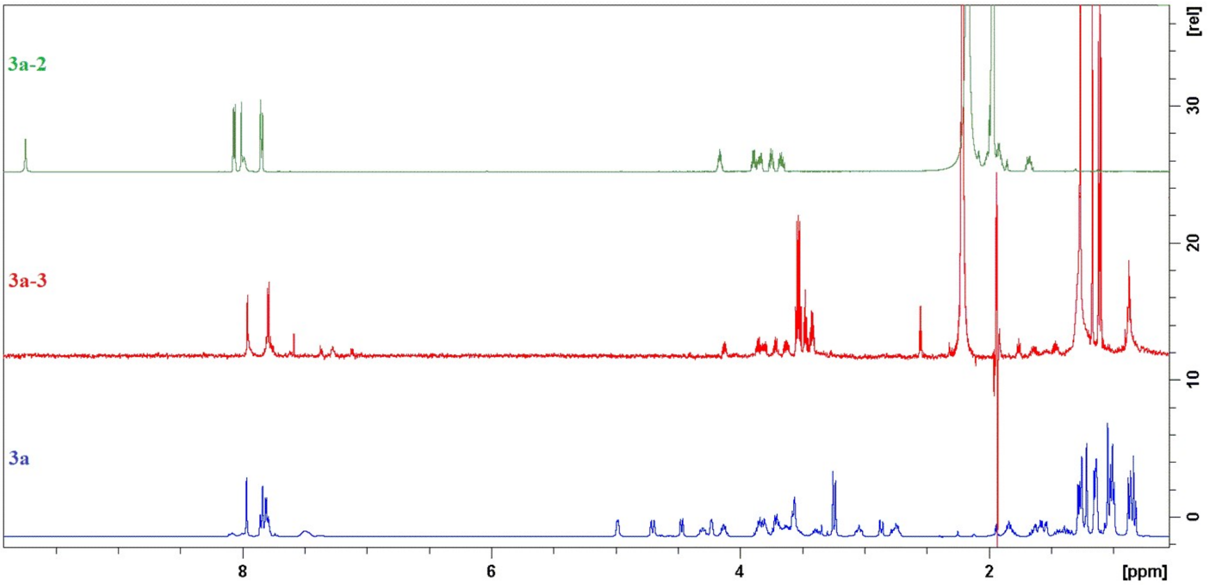

Figure S4. a) proton spectrum of the **3a-3** (signals from solvent impurities are marked with asterisk* and signals from ethanol are marked with double asterisk**), b) MS spectrum of the **3a-3** and c) overlaid proton spectra of the **3a**, **3a-2** and **3a-3**.

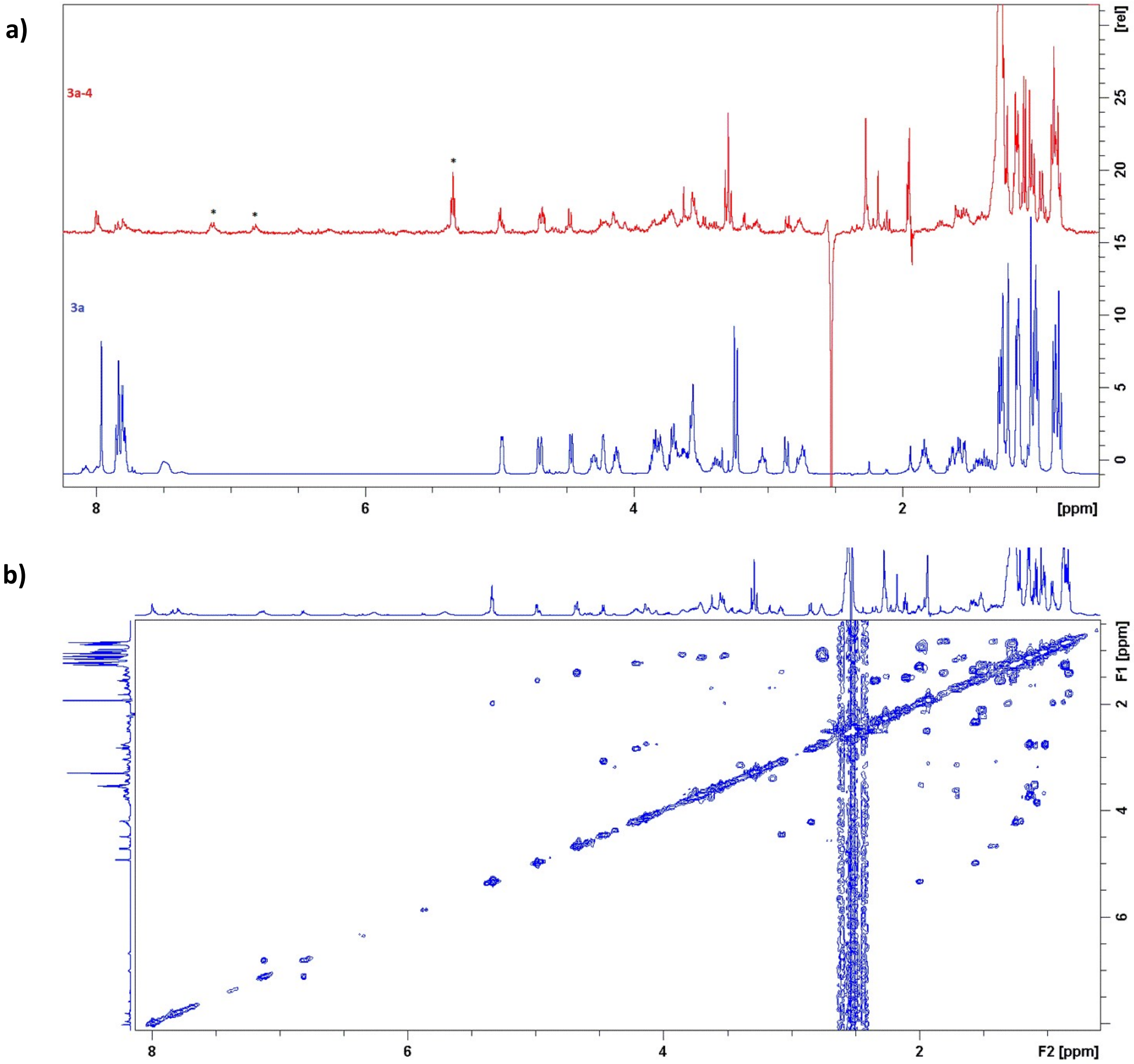
a)
3a-4
3a
[rel]
[ppm]
b)
F1 [ppm]
F2 [ppm]

c)

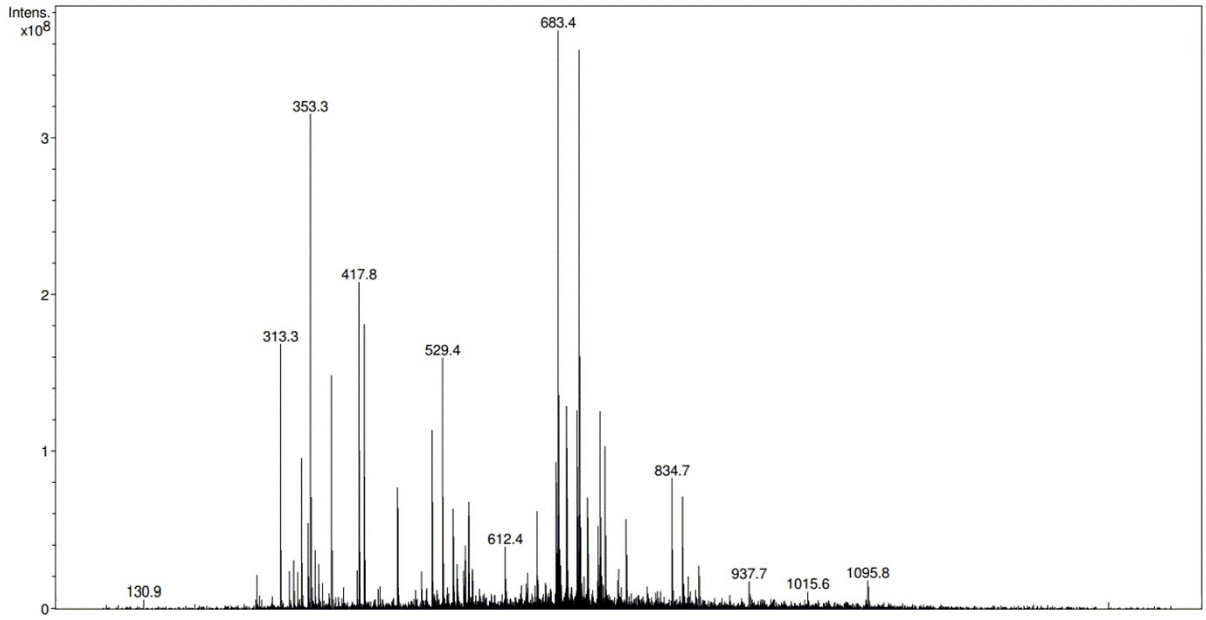

Figure S5. a) overlaid proton spectra of the compounds **3a** and **3a-4** (signals from solvent impurities are marked with asterisk*), b) COSY spectrum and c) MS spectrum of the compound **3a-4**.

a)
b)
c)

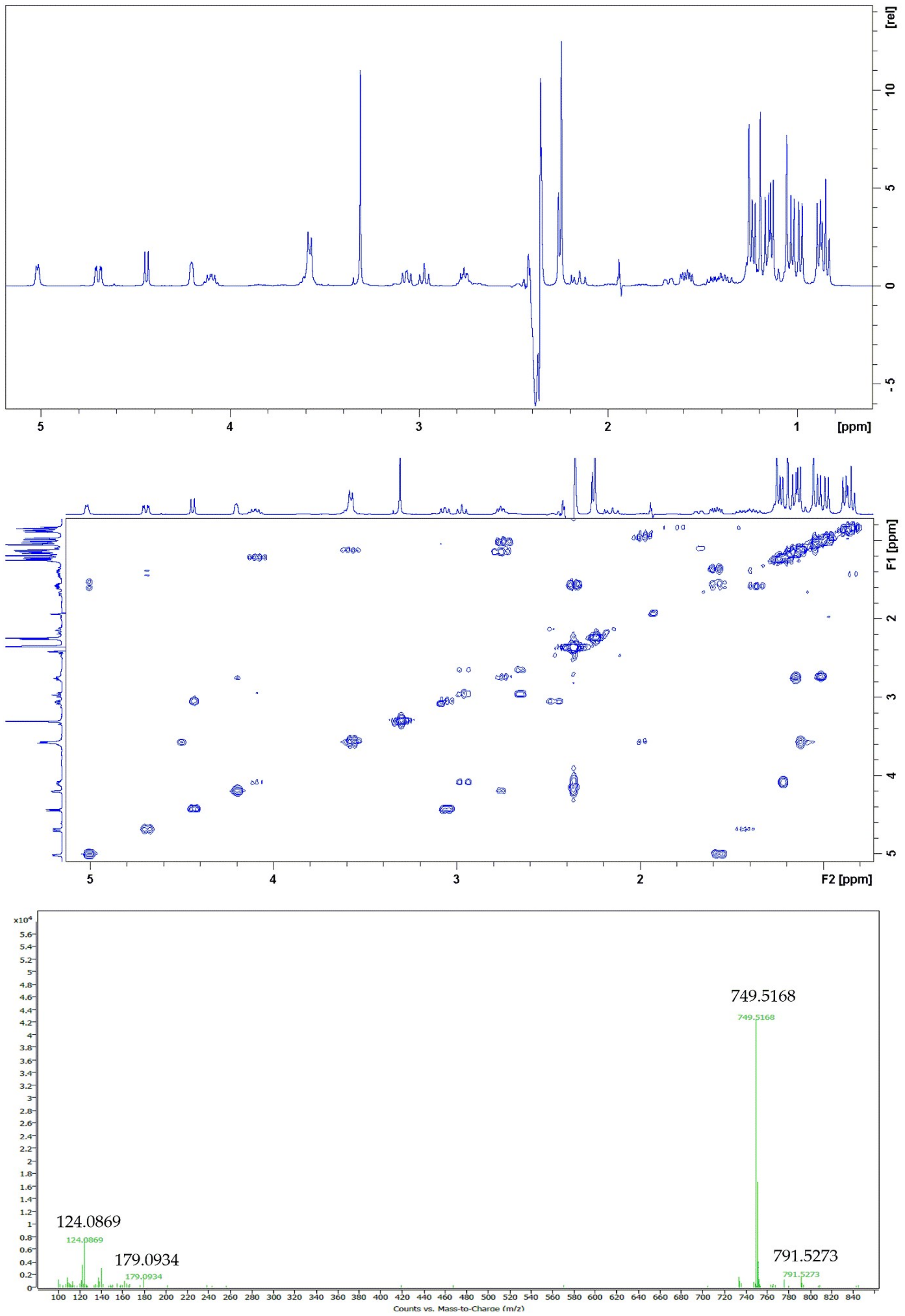

Figure S6. a) proton spectrum of the compound **3a-5,** b) COSY spectrum of the compound **3a-5** and c) MS spectrum of the compound **3a-5**.

a)

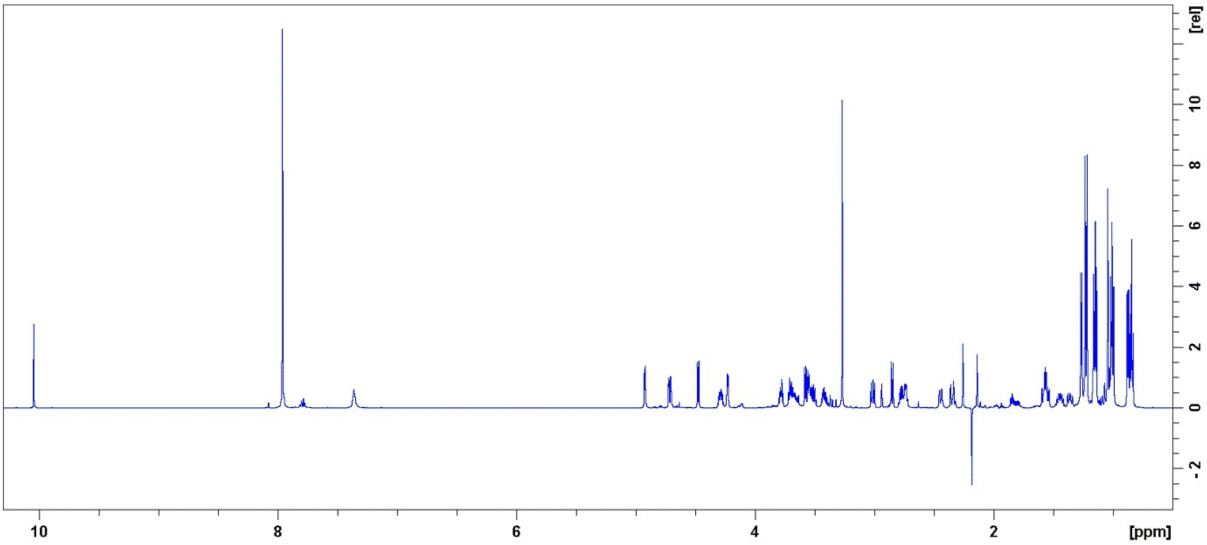
[rel]
10
8
6
4
2
0
-2
10
8
6
4
2
[ppm]

b)

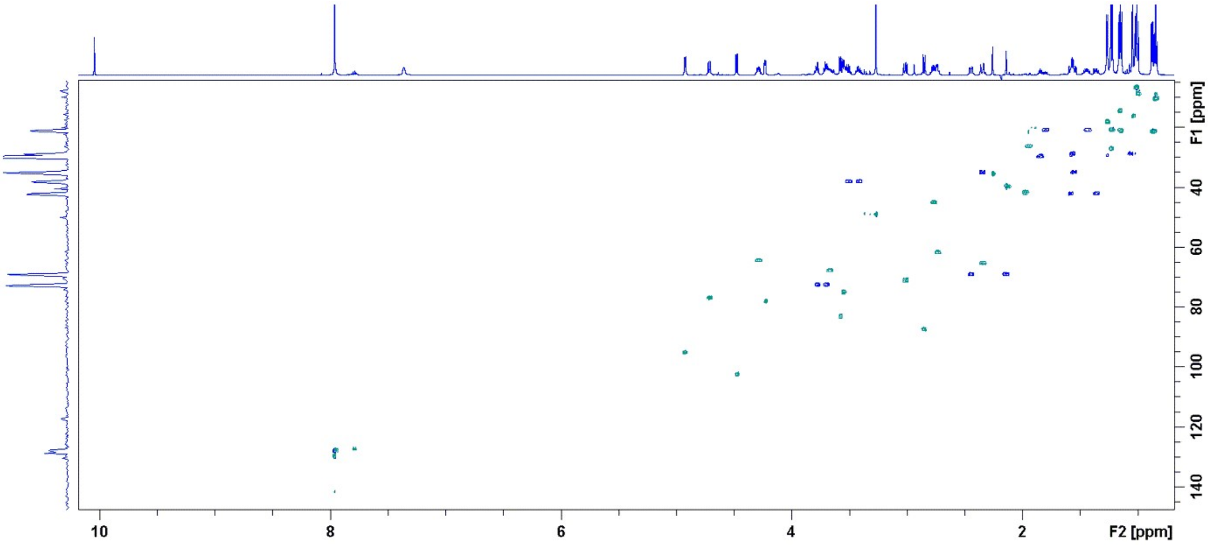
F1 [ppm]
20
40
60
80
100
120
140
10
8
6
4
2
F2 [ppm]

c)

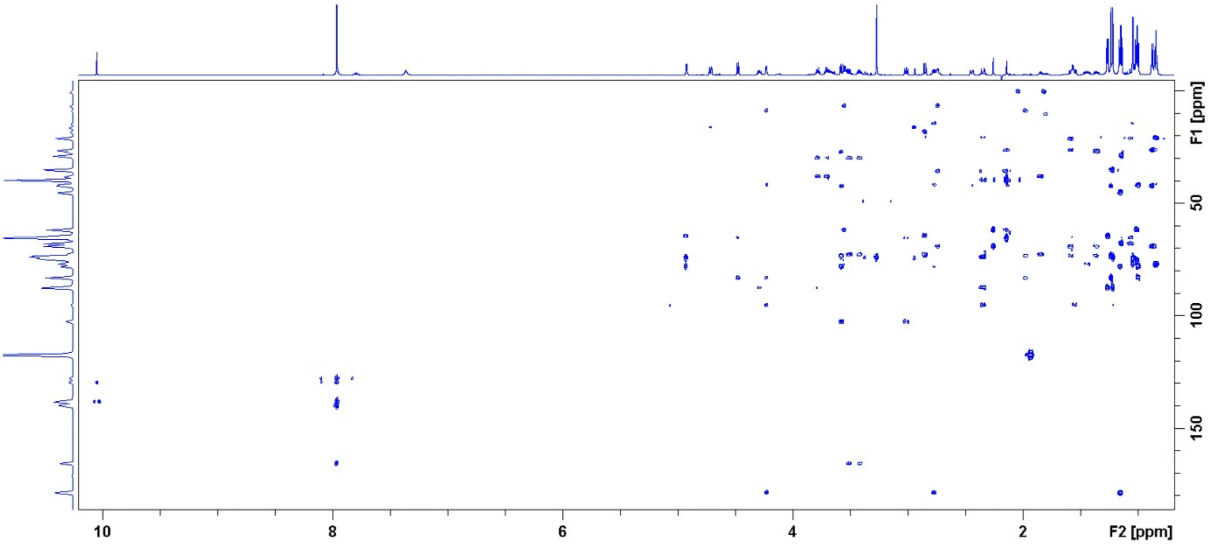
F1 [ppm]
50
100
150
10
8
6
4
2
F2 [ppm]

d)

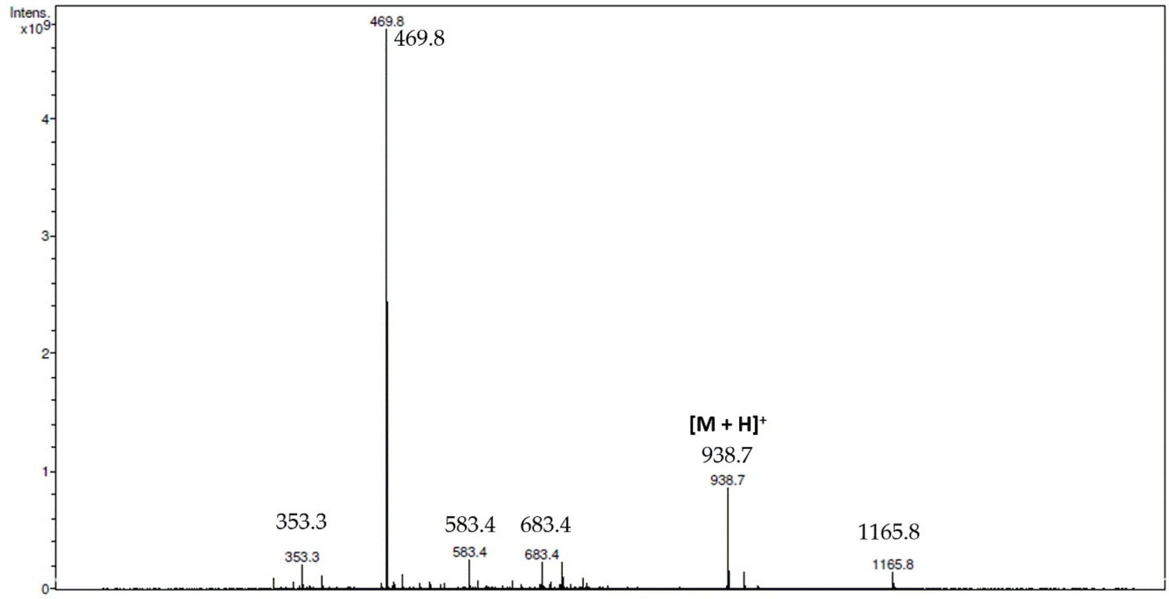

Figure S7. a) proton spectrum, b) HSQC spectrum, c) HMBC spectrum and d) MS spectrum of the compound **3a-6**.

a)

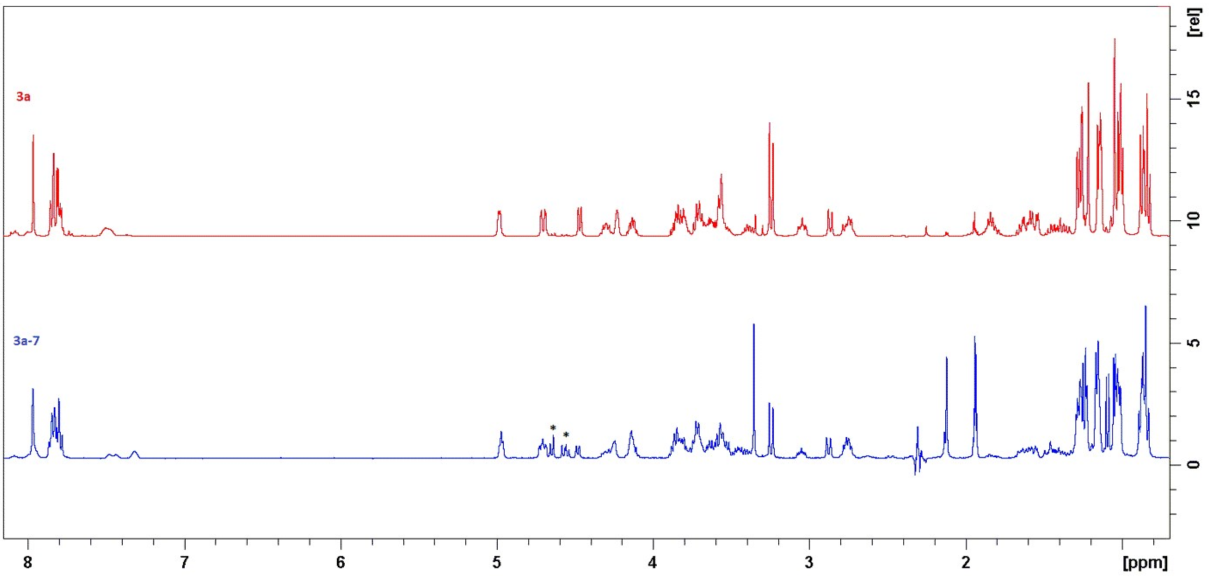

b)

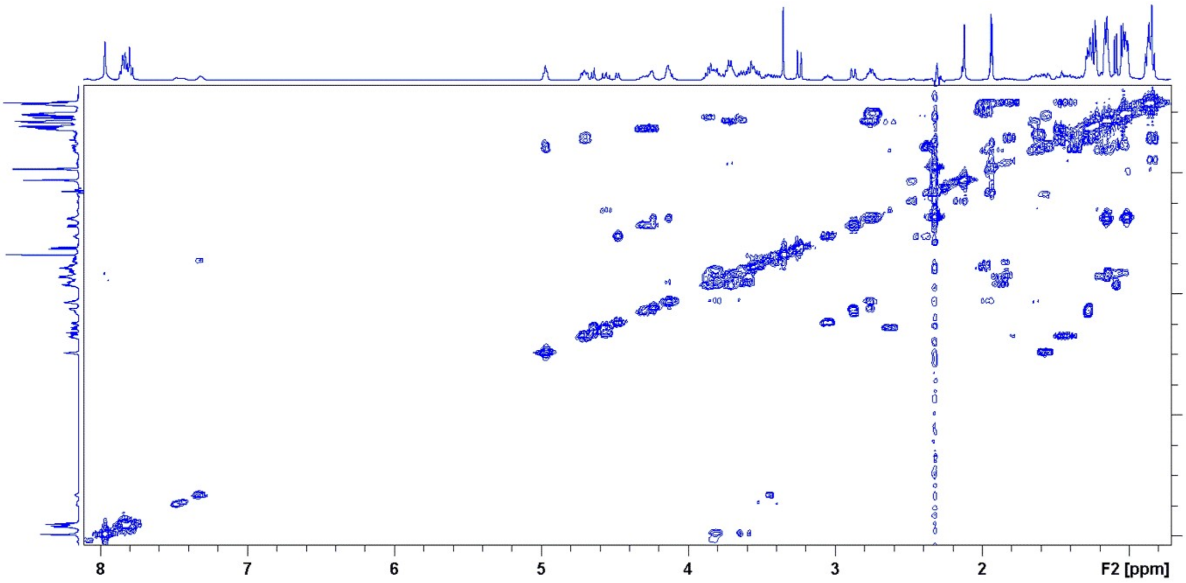
F1 [ppm]
2
4
6
8
7
6
5
4
3
2
F2 [ppm]

c)

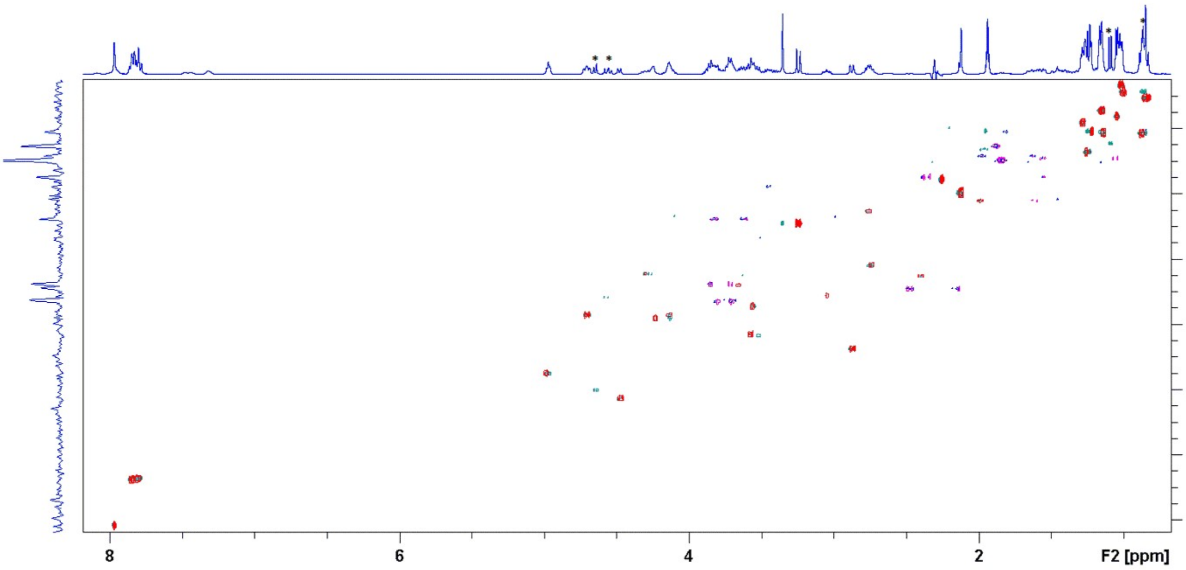
F1 [ppm]
20
40
60
80
100
120
8
6
4
2
F2 [ppm]

d)

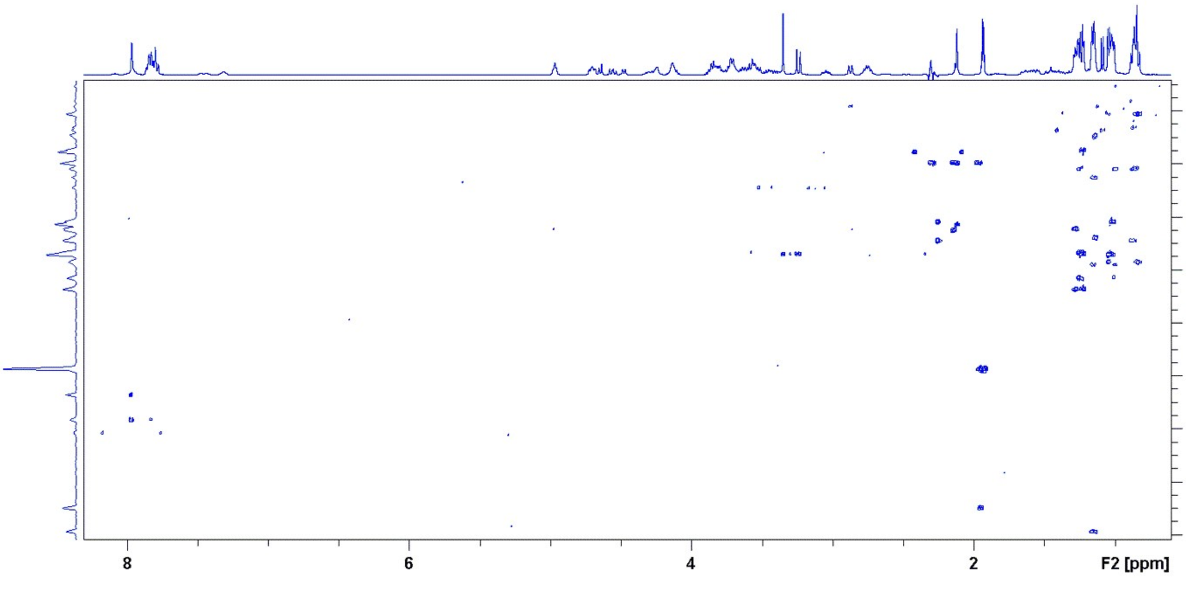
F1 [ppm]
20
40
60
80
100
120
140
160
8
6
4
2
F2 [ppm]

e)

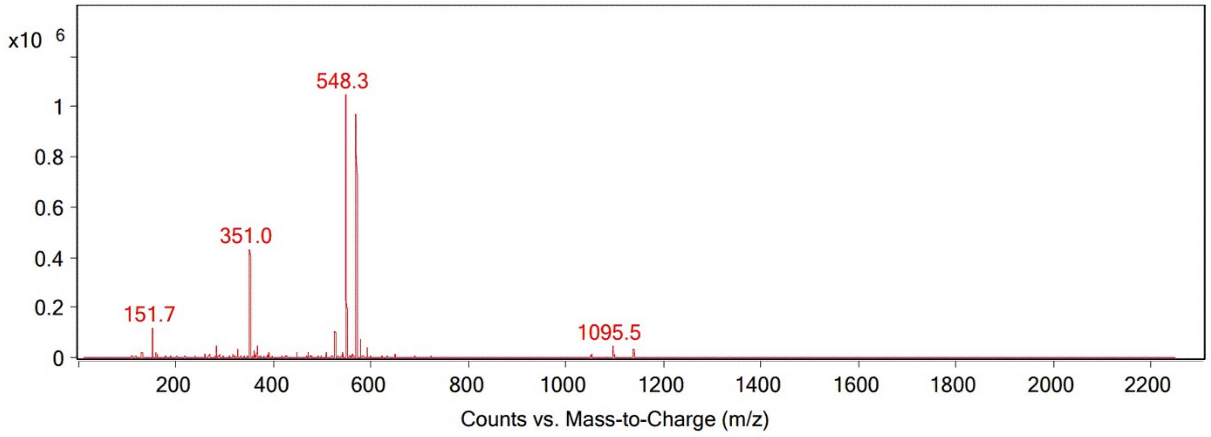

Figure S8. a) overlaid proton spectra of the **3a-7** and **3a**, b) COSY spectrum, c) overlaid HSQC spectrum of the **3a-7** and **3a** (blue and green contours belong to the **3a-7**, while red and pink contours belong to the **3a**; signals from unknown impurities are marked with asterisk*), d) HMBC spectrum and e) MS spectrum of the compound **3a-7**.

a)

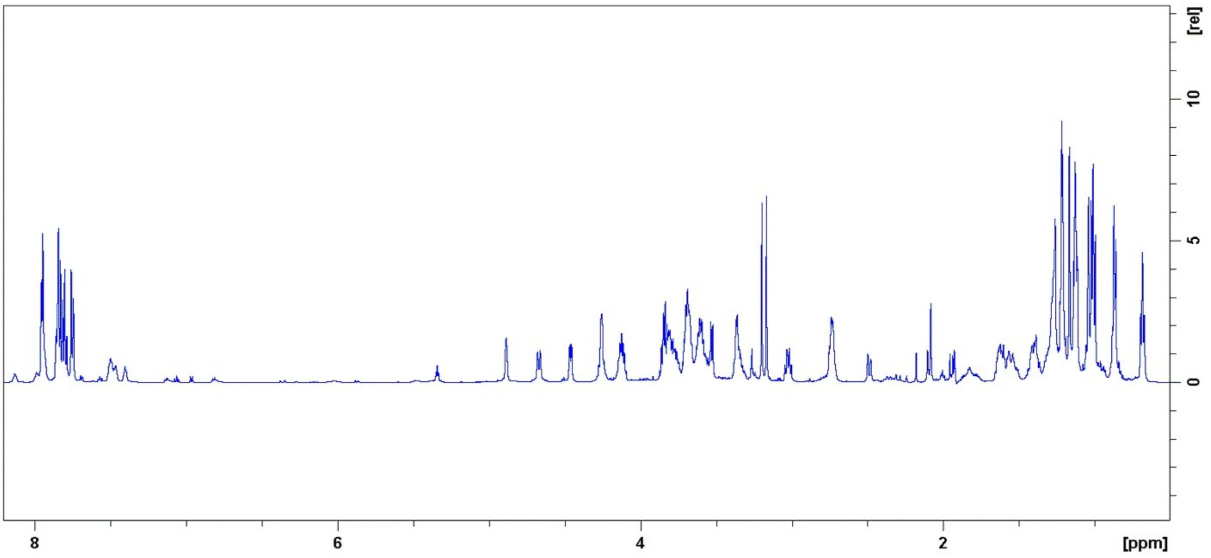

b)

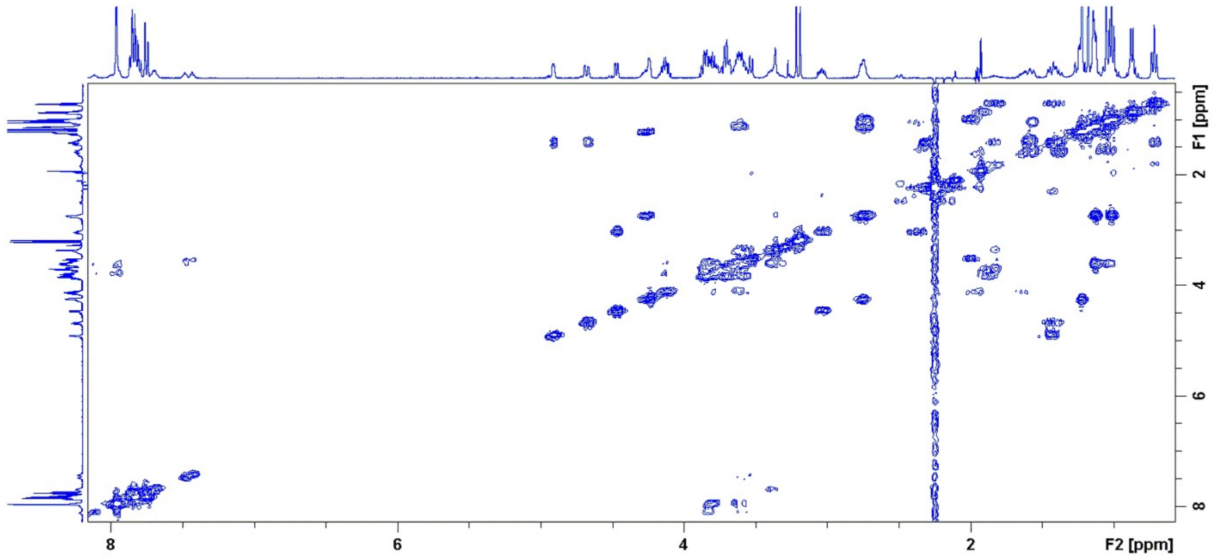

c)

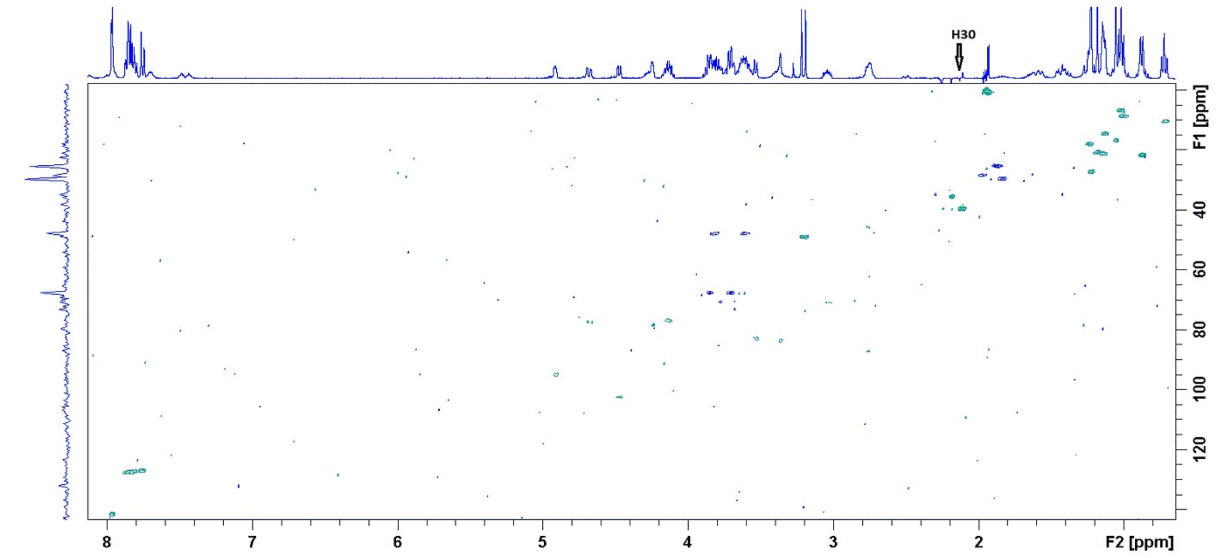

d)

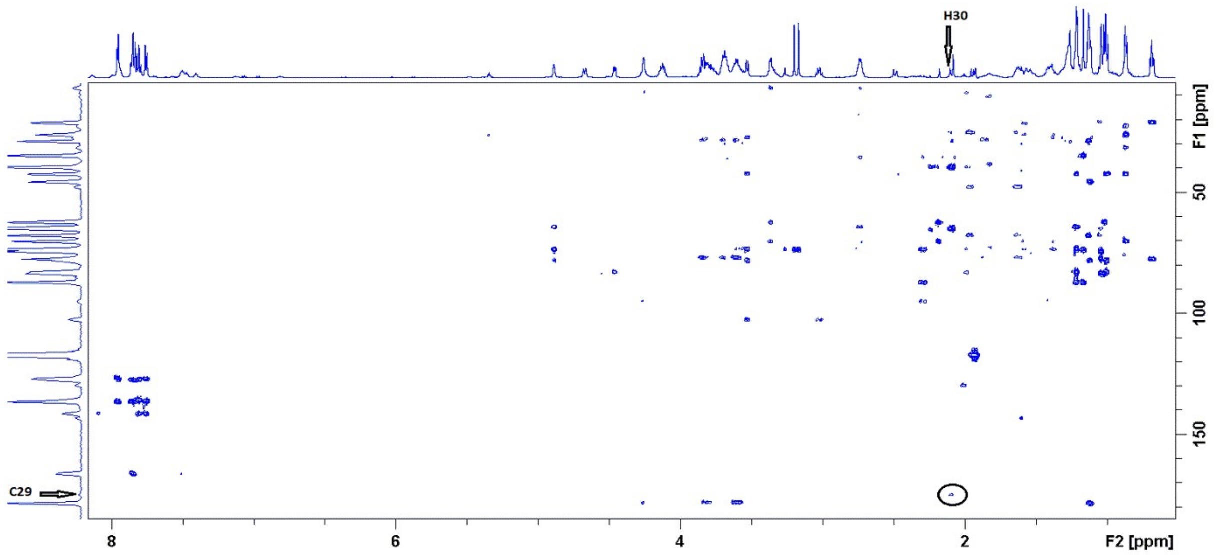

Figure S9. a) proton spectrum, b) COSY spectrum, c) HSQC spectrum, d) HMBC spectrum of the compound **3a-8**. HMBC correlation of a methyl group protons H30 with a carbonyl group C29 is marked in black.

Table S1. ^{1}H and ^{13}C chemical shifs of compound **3a-7**

**Compound**			**3a-7**		
**Atom**	**δ(^{1}H)/ppm**	**δ(^{13}C)/ppm**	**Atom**	**δ(^{1}H)/ppm**	**δ(^{13}C)/ppm**
**1**	-	178.8	**22**	7.97	141.6
**2**	2.76	45.3	**4″- 22a**	-[a]	-
**2Me**	1.16	14.38	**4″- 22b**	7.99	-
**3**	4.24	78.2	**23**	-	-[a]
**4**	1.98	41.83	**24**	3.82; 3.65	47.8

**4Me**	1.01	8.77	**25**	4.14	77.85
**5**	3.58	83.2	**26**	1.98; 1.64	28.35
**6**	-	73.5	**27**	1.89	25.45
**6Me**	1.25	27.23	**28**	3.86; 3.73	67.6
**6OH**	-[a]	-	**1'**	4.48	102.69
**7eq; 7ax**	1.46	41.6	**2'**	3.05	71.04
**8**	1.98	26.16	**2'OH**	-[a]	-
**8Me**	0.87	21.43	**3'**	2.40	65.05
**9eq; 9ax**	2.48; 2.15	69.0	**3'NMe$_2$**	2.12	39.72
**9a-N**	2.26	35.56	**4'eq; 4'ax**	1.57; 1.06	29.12
**10**	2.75	62.0	**5'**	3.66	67.9
**10Me**	1.02	6.63	**5'Me**	1.15	21.11
**11**	3.66	67.7	**1''**	4.97	95.0
**11OH**	-[a]	-	**2''eq; 2''ax**	2.37; 1.58	34.92
**12**	-	74.6	**3''**	-	74.1
**12Me**	1.05	16.17	**3''Me**	1.24	20.8
**12OH**	-[a]	-	**3''OMe**	3.25	49.1
**13**	4.71	77.1	**4''**	2.88	87.4
**14eq; 14ax**	1.82; 1.46	21.06	**4″- a**	3.82; 3.71	72.7
**14Me**	0.85	10.46	**4″- b**	1.85	29.73
**15**	-	-[a]	**4″- c**	3.45	37.87
**16**	-	135.64	**4″- d**	7.32	-
**17; 21**	7.84	127.5	**5''**	4.26	64.6
**18; 20**	7.81	127.2	**5''Me**	1.28	18.21
**19**	-	136.7			

-[a] not assigned
